# Isorhamnetin, Hispidulin, and Cirsimaritin Identified in *Tamarix ramosissima* Barks from Southern Xinjiang and Their Antioxidant and Antimicrobial Activities

**DOI:** 10.3390/molecules24030390

**Published:** 2019-01-22

**Authors:** Xiaopu Ren, Yingjie Bao, Yuxia Zhu, Shixin Liu, Zengqi Peng, Yawei Zhang, Guanghong Zhou

**Affiliations:** 1 Key Laboratory of Meat Processing and Quality Control, Ministry of Education China, Jiangsu Collaborative Innovation Center of Meat Production and Processing, Quality and Safety Control, College of Food Science and Technology, Nanjing Agricultural University, Nanjing 210095, China; alarxp@126.com (X.R.); 2015208017@njau.edu.cn (Y.B.); 2016208019@njau.edu.cn (Y.Z.); liushixin_004@163.com (S.L.); ghzhou@njau.edu.cn (G.Z.); 2 Xinjiang Production & Construction Group Key Laboratory of Agricultural Products Processing in Xinjiang South, College of Life Science, Tarim University, Alar 843300, China

**Keywords:** *Tamarix ramosissima*, polyphenolics, antioxidant activity, antimicrobial activity, isorhamnetin, hispidulin, cirsimaritin

## Abstract

As a natural potential resource, *Tamarix ramosissima* has been widely used as barbecue skewers for a good taste and unique flavor. The polyphenolics in the branch bark play a key role in the quality improvement. The purposes of the present work were to explore the polyphenolic composition of *T. ramosissima* bark extract and assess their potential antioxidant and antimicrobial activities. Hispidulin and cirsimaritin from *T. ramosissima* bark extract were first identified in the *Tamarix* genus reported with UPLC-MS analysis. Isorhamnetin (36.91 μg/mg extract), hispidulin (28.79 μg/mg extract) and cirsimaritin (13.35 μg/mg extract) are rich in the bark extract. The extract exhibited promising antioxidant activity with IC_50_ values of 117.05 μg/mL for 1,1-diphenyl-2-picrylhydrazyl (DPPH) and 151.57 μg/mL for hydroxyl radical scavenging activities, as well as excellent reducing power with an EC_50_ of 93.77 μg/mL. The bark extract showed appreciable antibacterial properties against foodborne pathogens. *Listeria monocytogenes* was the most sensitive microorganism with the lowest minimum inhibitory concentration (MIC) value of 5 mg/mL and minimum bactericidal concentration (MBC) value of 10 mg/mL followed by *S. castellani* and *S. aureus* among the tested bacteria. The *T. ramosissima* bark extract showed significantly stronger inhibitory activity against Gram-positive than Gram-negative bacteria. Nevertheless, this extract failed to show any activity against tested fungi. Overall, these results suggested that *T. ramosissima* shows potential in improving food quality due to its highly efficacious antioxidant and antibacterial properties.

## 1. Introduction

Various species of *Tamarix*, which are widely distributed throughout Europe, America, Asia, and Africa, have been used as herbal medicines in many civilizations due to the presence of polyphenolic compounds [1]. The methanolic extract of dried aerial components of *T. gallica* from India was found to prevent thioacetamide-promoted oxidative stress and toxicity and exhibited significant properties to reduce the susceptibility of the hepatic microsomal membrane to iron-ascorbate induced lipid peroxidation, H_2_O_2_ content, glutathione *S*-transferase, and xanthine oxidase activities in rats [2]. Yao et al. [3] showed that tamaractam, a new phenolic aromatic ring compound from *T. ramosissima* tender branches and leaves from the Ningxia province, displayed a strong inhibitory activity on cell proliferation in rheumatoid arthritis fibroblast-like synoviocytes, suggesting that it could remarkably induce cellular apoptosis and increase activated caspase-3/7 levels. Rahman et al [4] indicated that the methanolic extract of *T. indica* roots from Bangladesh exhibited excellent antinociceptive and anti-inflammatory properties. Significant writhing inhibition was produced by the extract in acetic acid-induced writhing in mice when comparable to the standard diclofenac sodium drug at the doses of 500 and 25 mg/kg body weight, respectively, and showed a significant anti-inflammatory activity against carrageenan-induced paw oedema in rats at oral doses of 200 and 400 mg/kg body weight compared to the standard drug aspirin. The wide spectrum of these medicinal properties may be mainly attributed to the presence of polyphenolic compounds in *Tamarix*, such as flavonoids and phenolic acids [1,2].

The leaves and flowers of *Tamarix* are rich in the polyphenolic compounds [5]. Sultanova et al. [1] identified tamarixetin in the leaves of *T. ramosissima* from southern Kazakhstan, and showed a high 1,1-diphenyl-2-picrylhydrazyl (DPPH) radical scavenging activity and antimicrobial activity against a number of pathogens, unambiguously specifying that the antioxidant and antibacterial activities of leaves were associated with the presence of polyphenolic compounds. Ksouri et al. [5] showed that syringic acid, isoquercetin and catechin were the major phenolics in the methanolic *T. gallica* leaf and flower extracts from south Tunis and that the flowers exhibited a higher antioxidant activity than that of the leaves, with IC_50_ values of the flower extracts being 1.3 (β-carotene bleaching) to 19-fold (lipid peroxidation inhibition) lower than those of leaves due to the higher total phenolic content (TPC). Meanwhile, the antibacterial properties of the leaf and flower methanolic extracts against human pathogen strains were also appreciable with a maximum inhibition zone of 15 mm against *Micrococcus luteus*. Unfortunately, there were few studies on the identification of polyphenolics, antioxidant and antimicrobial activities of the stem barks of *T. ramosissima* from southern Xinjiang.

*T. ramosissima* is one of the main constructive species in the Tarim River basin native to Northwestern China. In southern Xinjiang, for hundreds of years, green branches of *Tamarix* have been used as barbecue skewers for a good taste and unique flavor. And based on our previous study, the effective substances were largely concentrated in the bark of the green branches. In the present work, we aim to (i) identify and quantify the major polyphenols present in the green branches bark of *T. ramosissima*, and (ii) evaluate their antioxidant and antimicrobial activities against foodborne pathogens. We show that hispidulin and cirsimaritin are first identified in the *Tamarix* genus reported from *T. ramosissima* bark extract and the extract exhibits satisfying antioxidant and antimicrobial activities, which suggests *T. ramosissima* shows potential in improving food quality to promote health.

## 2. Results and Discussion

### 2.1. Content and Variety of Total Polyphenolics from T. ramosissima Barks

#### 2.1.1. TPC and Total Flavonoid Content (TFC)

Many polyphenolic compounds, which are those containing one or more aromatic ring with one or more hydroxyl groups, act as antioxidants in natural plants due to their redox properties [5]. Flavonoids, which are one group of polyphenolics, are secondary metabolites in plants and act as antioxidants [6]. Many types of polyphenols, such as flavonoids and phenolic acids, were reported in *Tamarix* species [1,2,7,8,9,10,11]. In this work, the TPC of *T. ramosissima* bark extract was 323.45 mg gallic acid equivalent (GAE)/g, and the TFC was 87.32 quercetin equivalent (QE)/g. When TPC and TFC of the bark extract were compared with the data available for the same genus, it was found that the *T. ramosissima* bark contained much higher values, as shown in Table 1. The differences between the *Tamarix* species were obvious, and the TPC of *T. ramosissima* was 323.45 mg GAE/g extract, which was 9.39, 2.39 and 1.62 times higher than those of *T. gallica* leaves, *T. gallica* flowers, and *T. aphylla* leaves, respectively. The TFC of *T. ramosissima* in this work was much higher than those of *T. gallica* leaves and flowers. Additionally, the TPC and TFC varied greatly among different organs, and the bark was a standout organ based on Table 1.

#### 2.1.2. Variety and Content of the Polyphenolics

**Figure 1:**
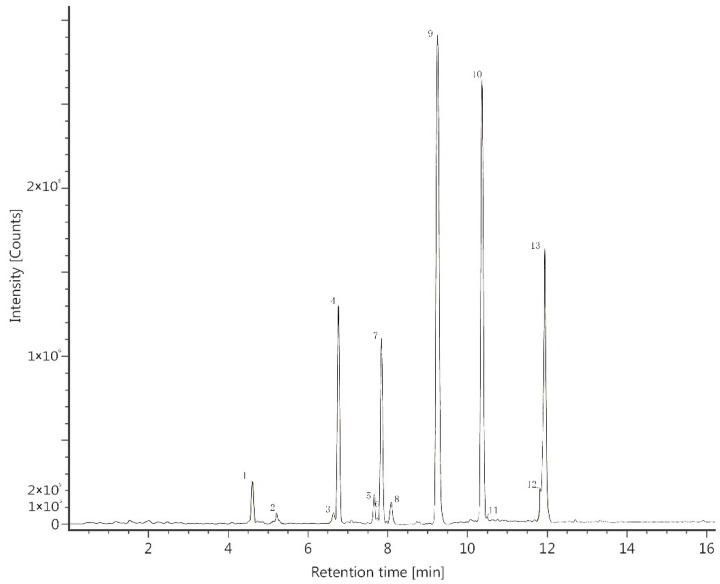
UPLC-MS chromatogram of polyphenolics in *T. ramosissima* crude extract infusion: quercetin 3-O-glucuronide (1), kaempferol 3-O-glucuronide (2), eriodictyol (3), quercetin (4), naringenin (5), tangeretin (6), kaempferol (7), hesperetin (8), isorhamnetin (9), hispidulin (10), apigenin (11), glycitein (12), cirsimaritin (13).

UPLC-MS data of the bark extract are shown in Table 2 and Figure 1. A total of 13 polyphenolic compounds were identified and, for the first time, hispidulin and cirsimaritin were isolated from the genus *Tamarix*: they are active ingredients in a number of traditional Chinese herbs [12,13]. Regarding the other main polyphenolics, isorhamnetin was reported to have been identified from *T. hispida*, *T. elongata* and *T. laxa* which were all collected from southern Kazakhstan [11,14]. Quercetin was a common compound in the *Tamarix* species and had been identified by several researchers [5,8,10,11,15]. Compared to the results of this work, other researchers obtained different polyphenolic compounds from *Tamarix*. Sultanova et al. identified tamarixetin from *T. ramosissima* leaves in Kazakhstan [1] and isolated rhamnocitrin, isorhamnetin and a pentacyclic triterpenoid from the aerial components of *T. hispida* [14]. Ksouri et al. [5] identified polyphenolics from *T. gallica* leaves and flowers in Tunis, and his results showed that the flower polyphenolics consisted of seven phenolic acids (gallic, sinapic, chlorogenic, syringic, vanillic, *p*-coumaric, and *trans*-cinnamic acids), six flavonoids ((+)-catechin, isoquercetin, quercetin, apigenin, amentoflavone, and flavone), 12 phenolic compounds including gallic, sinapic, chlorogenic, syringic, vanillic, rosmarinic, *p*-coumaric, ferulic, and *trans*-cinnamic acids, as well as two flavonoids (quercetin and amentoflavone) which were identified from the leaves. Yao et al [3] identified only three compounds, tamaractam, *cis*-*N*-feruloyl-3-*O*-methyldopamine and *trans-N*-feruloyl-3-*O*-methyl- dopamine from *T. ramosissima* in Yinchuan, China.

Meanwhile, a further 4 polyphenolic compounds in high amounts, isorhamnetin, hispidulin, cirsimaritin, and quercetin, were quantified in this work (Table 3). However, no literature was found to quantify the concentration of polyphenolics from *Tamarix*.

### 2.2. Antioxidant Activity of the Bark Extract

#### 2.2.1. DPPH Scavenging Activity

**Figure 2:**
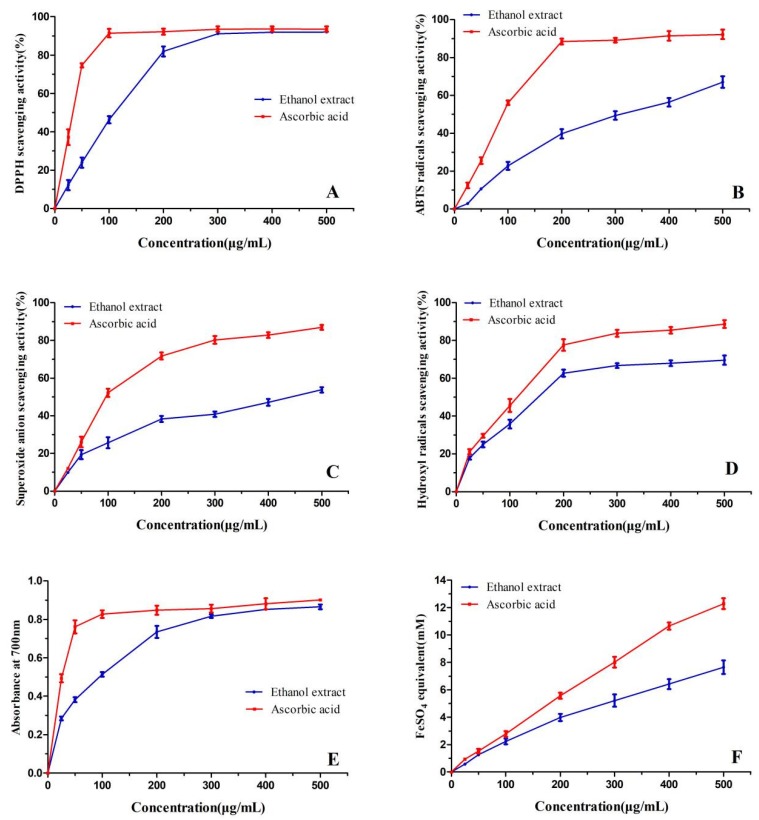
Scavenging activities on 1,1-diphenyl-2-picrylhydrazyl (DPPH) (**A**), 2,2′-Azinobis-(3-ethylbenzthiazoline-6-sulphonate) (ABTS) (**B**), superoxide radicals (**C**), hydroxyl radicals (**D**), reducing power (**E**) and ferric reducing antioxidant power (FRAP) (**F**) of the bark extract of *T. ramosissima* and ascorbic acid. Data are presented as means ± SD of triplicates.

The DPPH free radicals have been extensively used to investigate the scavenging activity of natural antioxidants. Figure 2A shows the results of the scavenging DPPH radical activity of *T. ramosissima* bark extracts. The scavenging activity increased sharply when the concentration increased from 25 to 200 μg/mL and trended towards a plateau after 300 μg/mL (91.70% ± 0.78), at which maximal scavenging activity on the DPPH radicals was reached. There was no significant difference of the scavenging activity between the bark extract groups and the control (ascorbic acid) (*p* > 0.05) at concentrations of more than 200 μg/mL. There was also no significant difference among the concentrations of 300, 400, and 500 μg/mL of the bark extract groups (*p* > 0.05). The IC_50_ value (117.05 μg/mL) of *T. ramosissima* bark extract for scavenging activity against DPPH was much higher than the results of Ksouri et al. [5], who found that the IC_50_ values on the DPPH radical of the *T. gallica* flower and leaf extracts were 2 and 9 μg/mL, respectively, due to the structural conformation of the antioxidants. In the main polyphenols of the *T. gallica* flower and leaf extracts, the second hydroxyl group of gallic, chlorogenic, and rosmarinic acid and catechin were in the ortho position, while the compounds of sinapic, syringic, vanillic, and ferulic acid had ortho-methoxy substitutions. These compounds in the flower and leaf extracts included a second hydroxyl group in the ortho or para position, and the ortho-methoxy substitution group increased antioxidant efficiencies [16]. However, isorhamnetin and hispidulin in the bark extract had the second hydroxyl group in the meta position. This is potentially the reason for the lower scavenging activity on the DPPH radicals compared to the results of Ksouri et al. [5].

#### 2.2.2. 2,2′-Azinobis-(3-ethylbenzthiazoline-6-sulphonate) (ABTS) Scavenging Activity

The scavenging activity of 2,2′-Azinobis-(3-ethylbenzthiazoline-6-sulphonate) (ABTS) radicals is another widely used method to assess the radical scavenging capacity of natural antioxidants [17]. As shown in Figure 2B, the ABTS radical scavenging activity of the *T. ramosissima* bark extract increased with the sample concentration. The scavenging activity of the polyphenolics on the ABTS· was correlated with their concentrations [18]. At a concentration of 500 μg/mL, the ABTS radical scavenging activity of the bark extract was 67.02%. The scavenging activities of bark extract groups from 25 to 500 μg/mL were significant lower than those of the control (*p* < 0.05). Additionally, there were significant differences in the ABTS scavenging activity among the bark extract concentrations (*p* < 0.05). The IC_50_ value of the bark extract on the ABTS radicals (314.88 μg/mL) was considerably higher than that of ascorbic acid (82.90 μg/mL), similar to the IC_50_ of the *T. gallica* flower extract (316.7 μg/mL), and 3.3 times lower than that of the *T. gallica* leaf extract [5]. The decreased IC_50_ of the *T. ramosissima* bark extract and *T. gallica* flower extract were probably because of the increased level of TPC and TFC (Table 1). Moreover, the difference in polyphenolic components among the extracts may be one of the reasons for the different scavenging activity on the ABTS radicals.

#### 2.2.3. Superoxide Anion Scavenging Activity

According to Yagi [19], as opposed to the mechanism of DPPH and ABTS radical reactions, the mechanism of superoxide and hydroxyl radicals was peroxide decomposition. The results of scavenging activity on superoxide radicals of the bark extract are presented in Figure 2C. The scavenging activity on the superoxide radical of the bark extract increased for concentrations ranging from 0 to 500 μg/mL. At the concentrations lower than 50 μg/mL of bark extract, there was no significant difference in scavenging activity of superoxide radicals between the bark extract and the control group. The difference in scavenging activity on superoxide radical between the concentrations of 200 and 300 μg/mL of bark extract was not significant. The IC_50_ value of the bark extract for the superoxide radical was 442.53 μg/mL, much higher than that of the results of Ksouri et al. [5], who found that the IC_50_ on the scavenging activity of the *T. gallica* flower and leaf extracts were 3 and 22 μg/mL, respectively. In the *T. gallica* flower and leaf extracts, hydroxyl groups were located at the 3′- and 4′-positions of the B-ring in quercetin, catechin, and isoquercetin that exhibited much higher scavenging activity on superoxide and hydroxyl radicals [19,20]. In the present work, isorhamnetin, hispidulin, and cirsimaritin in the bark extract possessed only one single hydroxyl group at the 4′-position of the B-ring, which exhibited slight scavenging activity.

#### 2.2.4. Hydroxyl Radicals Scavenging Activity

As Figure 2D shows, the scavenging activities on hydroxyl radicals increased with the concentrations. The hydroxyl radical scavenging activity increased rapidly in the range of 25 to 200 μg/mL, before reaching a plateau from 300 to 500 μg/mL. Significant differences in the activity were found among the concentrations of 25, 50, 100, and 200 μg/mL of the bark extracts (*p* < 0.05). The scavenging activities of the bark extract in these concentrations were not significantly different from the ascorbic acid (*p* > 0.05). The IC_50_ of the bark extract on the hydroxyl radical was 151.57 μg/mL, which was not significantly higher than that of the ascorbic acid (114.08 μg/mL), suggesting a satisfactory hydroxyl radical scavenging activity. It was evident from Figure 2C,D that the scavenging activity of the bark extract on hydroxyl radicals was stronger than on superoxide radicals. At 200 μg/mL, the bark extract quenched 62.66% of hydroxyl radicals while only quenching 38.28% of superoxide radicals. The stronger scavenging activity on hydroxyl radicals of the bark extract than on superoxide radicals may be associated with the carbonyl function at the C-4 position in the structures of isorhamnetin, hispidulin, and cirsimaritin. The result was in accordance with Husain et al., who found that the presence of a carbonyl functional group at the C-4 position played an important role in the hydroxyl radical quenching ability and that quercetin possessing a carbonyl showed higher quenching ability on the hydroxyl radicals than the carbonyl-devoid catechin [20].

#### 2.2.5. Reducing Power

The reducing power of natural antioxidants, which was determined using a modified Fe^3+^ to Fe^2+^ reduction assay, has been declared to be associated with their antioxidant activity [21]. As shown in Figure 2E, the reducing power of *T. ramosissima* bark extract was excellent and increased with the amount of the extract. The reducing power of bark extract at concentrations below 200 μg/mL increased rapidly with concentrations but was significantly lower compared with the control (*p* < 0.05). At the concentration of 300 μg/mL, the reducing power of the bark extract was similar to that of ascorbic acid (*p* > 0.05). Furthermore, there were also no significant differences among the concentrations of 300, 400, and 500 μg/mL of the bark extract groups (*p* > 0.05). The EC_50_ of the bark extract was 93.77 μg/mL, similar to the EC_50_ (76.67 μg/mL) of *T. gallica* leaf extract [5]. However, in another report of Ksouri et al. [22] on *T. gallica* flower and leaf extracts from a different location, the EC_50_ values of the reducing power were 84.3 and 205 μg/mL, respectively.

#### 2.2.6. Ferric Reducing Antioxidant Power (FRAP)

The mechanism of the ferric reducing antioxidant power (FRAP) assay was similar to the reducing power, both of which relied on the ability of antioxidants to reduce iron (III) to iron (II). More specifically, in the FRAP assay, a ferric tripyridyltriazine (FeIII-TPTZ) complex was reduced to the ferrous form (FeII-TPTZ) at low pH value, and several researchers considered the assay as the total antioxidant power [23,24]. In the present work, the trends for ferric ion reducing activities of *T. ramosissima* bark extract and ascorbic acid are shown in Figure 2F. For both of them, the FeSO_4_ equivalent (mM/g) clearly increased due to the formation of the Fe^2+^-TPTZ complex with increasing concentration. The FeSO_4_ equivalent increased linearly with the concentrations (R^2^ = 0.9879 for the bark extract, 0.9961 for ascorbic acid). There was no significant difference between the bark extract group and the control at concentrations ranging from 0 to 100 μg/mL (*p* > 0.05). However, the differences became larger with increasing concentrations. At the concentration of 500 μg/mL, the FeSO_4_ equivalent was 7.64 mM/g for the bark extract and 12.28 mM/g for the positive control, both of which were higher than those of *Barringtonia racemosa* and kudingcha crude extracts determined by the same methods [25,26]. Hidalgo et al. [27] found that in the FRAP assay, the polyphenols with 3-hydroxyl group in the C-ring (such as isorhamnetin and quercetin in the bark extract of the present work) showed high antioxidant power and that the antioxidant activity did not decrease when the 3-hydroxyl group in the C-ring was blocked if the 3′,4′-dihydroxy structure in the B-ring was retained (as in the case of quercetin 3-O-glucuronide in the bark extract of the present work).

As previously mentioned, the antioxidant activity of the bark extract of *T. ramosissima* was satisfactory and the presence of polyphenolics, especially flavonoids, probably played a significant role. The findings of Ksouri et al. [5] largely supported the claims of the present work, which suggests that the extracts of *T. gallica* showed very high antioxidant activity due to the presence of polyphenolics and that the antioxidant properties exhibit a high positive correlation with polyphenolic content. Sultanova et al. [1] also specifically stated that the antioxidant activity was associated with the presence of polyphenolic substances. In the bark extract of *T. ramosissima*, different polyphenols exhibited various antioxidant activities. Isorhamnetin, a 3′-*O*-methylated metabolite of quercetin, exerts excellent antioxidant effects, which reduces oxidative stress due to free radicals by induction of NF-E2-related factor 2 (Nrf2)-dependent antioxidant genes [28]. Cirsimaritin is a small molecular natural flavonoid, mainly derived from the medicinal plant Herba Artemisiae Scopariae, which exhibits a variety of beneficial activities including antioxidant activity [29]. Quercetin is the typical flavonoid structure and is widely used as a nutritional supplement due to its antioxidant and anti-inflammatory properties [30]. Hispidulin, also named 6-methoxy-5,7,4′-trihydroxyflavone, has been shown to possess anti-inflammatory and antioxidative activities [31], although the antioxidant activity of hispidulin is very weak compared with quercetin [32]. All of these polyphenolic compounds contributed to the antioxidant activity of the *T. ramosissima* bark extract. In general, the antioxidant property of a given compound is thought to be closely linked to its structural features, including the ortho-dihydroxy structure in the B-ring, the 2,3 double bond in conjugation with a 4-oxo function, the presence of the 3- and 5-OH functions and glycosidic moieties and the number and position of hydroxyl and methoxy groups [27]. Moreover, the nature of the radical and its specific reaction mechanism also exert great influence on the antioxidant activity of the tested polyphenolics. All of these elements determine the final effects of the polyphenolics.

### 2.3. Antimicrobial Activity

The antimicrobial activities of *T. ramosissima* bark extract at different concentrations are shown in Table 4. For the Gram-positive bacteria, the inhibition zone against *Listeria monocytogenes* at the concentration of 10 mg/mL of the bark extract was the largest (*p* < 0.05). The inhibition zones against *L. monocytogenes* and *Staphylococcus aureus* were larger than that of *Bacillus cereus* at the concentration of 5 mg/mL, and significantly increased with the concentrations (*p* < 0.05). However, the inhibition zone against *S. aureus* was not influenced by the concentration of the bark extract. Among these Gram-positive strains, *L. monocytogenes* was the most sensitive microorganism with the lowest minimum bactericidal concentration (MBC) values of 10 mg/mL. Excellent antibacterial activity against Gram-positive bacteria was also supported by Ksouri et al. [5], who found that the inhibition zone ranged from 7.00 to 15.00 mm at the concentrations of 2, 4 and 100 mg/mL of the *T. gallica* leaf and flower extracts. For *S. aureus*, the bark extract in this work showed much larger inhibition zones compared with the *T. gallica* leaf and flower extracts. The methanol extract of *T. indica* from Bangladesh also exhibited similar antibacterial activity against *S. aureus* with an inhibition zone of 10.80 mm [4]. Nevertheless, the *T. ramosissima* leaf extract from southern Kazakhstan showed no antibacterial activity against *S. aureus* and *B. cereus* [1]. The inhibitory effects of the bark extract on bacterial pathogens could be attributed to the isorhamnetin, hispidulin, or cirsimaritin in the *T. ramosissima* bark extract, of which the purified hispidulin showed antibacterial activity against *B. subtilis* with minimum inhibitory concentration (MIC) values of 50 μg/mL and *S. aureus* with MIC values of 100 μg/mL [33].

The inhibition zones against Gram-negative bacteria were increased with the concentrations (*p* < 0.05), except in the case of *Shigella castellani*. In the Gram-negative bacteria, the inhibition zones against *S. castellani* were the largest (*p* < 0.05) at the concentrations of 1 or 5 mg/mL of the bark extract. The inhibition zones against *Escherichia coli* and *S. castellani* were larger than those of *Pseudomonas aeruginosa* and *Salmonella typhimurium* at the concentration of 10 mg/mL (*p* < 0.05). *S. castellani* was the most sensitive to the bark extract, with the lowest MIC of 5 mg/mL and MBC values of 15 mg/mL. These results were consistent with those of Rahman et al., who found that the *T. indica* extract exhibited the largest inhibition zones against *S. sonnie* [4]. In the present work, *E. coli* was the second most sensitive to the bark extract with a MIC of 10 mg/mL and MBC values of 25 mg/mL. The *T. gallica* leaf and flower extracts from Tunis also had inhibitory effects on *E. coli* [5].

Compared to Gram-negative bacteria, Gram-positive bacteria were sensitive to the bark extract, with the mean of inhibition zones significantly larger at the concentrations of 1, 5 and 10 mg/mL (*p* < 0.05). Similar results can also be observed from the MIC values of these bacteria. These differences in inhibitory effects of the bark extract between Gram-negative bacteria and Gram-positive bacteria may be associated with the different cell wall components of the bacteria [34]. The antibacterial activity of the *T. ramosissima* bark extract was lower than that of gentamycin (*p* < 0.05). The *T. ramosissima* bark extract failed to show any activity against four tested fungi. The results of this work suggested that there may be a huge potential application of *T. ramosissima* to prevent the growth of foodborne pathogens.

## 3. Materials and Methods

### 3.1. Plant Materials and Extraction Procedure

Fresh twigs of *T. ramosissima* were collected from the Tarim River basin on the edge of the Taklamakan desert in Xinjiang (40°25′28.81″ N, 81°14′18.14″ E, Southwest of Alar city) in June 2017. The plant was identified by an expert taxonomist, Mr. Pang, who had been engaged on taxonomic research of *Tamarix* in South Xinjiang for a significant length of time at Tarim University. The peeled bark off green branches were air-dried and milled to a fine powder (mesh size: 1 mm). Approximately 500 g of powdered barks were soaked in 2500 mL of 60% ethanol for 40 min. Ultrasonic-assisted extraction was used, and the power was 600 W (VCX 750, Sonics, USA). The extracts were filtered through a Whatman no. 4 filter paper and evaporated under vacuum. Then, the vacuum freeze dryer (LGJ-10C, Four-ring, China) was employed to obtain the ethanol extracts and they were stored at −20 °C until analysis. All solvents and reagents were of analytical grade.

### 3.2. Determination of Total Polyphenolic Content

The TPC in the *T. ramosissima* bark extract was determined according to the Folin–Ciocalteu procedure as described by Jayasinghe et al [35] with some modifications, using gallic acid as a standard. 0.05 g of the bark extract was dissolved in 2 mL of ethanol-water solution and mixed with 2 mL of Folin–Ciocalteu’s phenol reagent. The mixture was kept for 3 min at room temperature, and then 2 mL of Na_2_CO_3_ solution (10%, *w*/*v*) was added to the mixture. The reaction mixture was allowed 1 h to incubate at ambient temperature in the dark, and the absorbance was then read at 530 nm. Deionized water was used as a blank sample. A standard calibration curve of gallic acid (0–0.4 mg/mL) was plotted (R^2^ = 0.9995). The results were expressed as milligrams of gallic acid equivalent per gram of the bark extract (mg GAE/g). All analyses were performed in triplicate.

### 3.3. Determination of Total Flavonoid Content

The TFC in the *T. ramosissima* bark extract was determined using the aluminum chloride colorimetric method as described by Chang et al [36] with some modifications using quercetin as a standard. In this experiment, 0.05 g of the bark extract was dissolved in 2 mL of 80% ethanol and then separately mixed with 0.1 mL of 10% aluminum chloride, 0.1 mL of 1 M potassium acetate and 2.8 mL deionized water. The reaction mixture was incubated at room temperature for 30 min, and the absorbance was then measured at 415 nm. Deionized water was used as a blank sample. A standard calibration curve of quercetin (0–0.1 mg/mL) was plotted (R^2^ = 0.9991). The concentration of flavonoid was expressed as milligrams of quercetin equivalent per gram of extract (mg QE/g). All analyses were performed in triplicate.

### 3.4. Analysis of Bark Extract by UPLC-MS

The samples were analyzed using a UPLC-MS following a method previously published, with some modifications [37]. The samples were analyzed by an LC-MS system (G2-XS QTof, Waters). First, 2 μL solutions were injected into the UPLC column (2.1 mm × 100 mm ACQUITY UPLC BEH C18 column containing 1.7 μm particles) with a flow rate of 0.4 mL/min. Buffer A consisted of 0.1% formic acid in water, and buffer B consisted of 0.1% formic acid in acetonitrile. The gradient was 5% Buffer B for 0.5 min, 5–95% Buffer B for 11 min, and 95% Buffer B for 2 min. Mass spectrometry was performed using an electrospray source in negative ion mode with the MSe acquisition mode and a selected mass range of 50–1200 *m*/*z*. The lock mass option was enabled using leucine-enkephalin (*m*/*z* 554.2615) for recalibration. The ionization parameters were the following: capillary voltage was 2.5 kV, collision energy was 40 eV, source temperature was 120 °C, and desolvation gas temperature was 400 °C. For quantification purposes, the four major compounds, isorhamnetin, hispidulin, cirsimaritin, and quercetin (Sigma, USA) were quantified using the calibration curves of their corresponding standards. Data acquisition and processing were performed using Masslynx 4.1 and results were presented as μg/mg extract.

### 3.5. Determination of Antioxidant Assays

#### 3.5.1. DPPH Radical Scavenging Activity

The DPPH free radical scavenging activity of the *T. ramosissima* bark extract was conducted according to the method of Yang et al [38], with some modifications. 0.5 mL of the bark extract solution at various concentrations (25, 50, 100, 200, 300, 400, and 500 μg/mL) was mixed with 3.5 mL of freshly prepared DPPH-ethanol solution (1 × 10^−4^ mol/L). The reaction mixture was mixed vigorously for 15 s and then kept in the dark for 30 min at room temperature. The absorbance was then measured at 517 nm, and the DPPH free radical scavenging percentage was calculated based on the following equation:

DPPH radical scavenging activity (%) = [1 − (A_1_ − A_2_)/A_0_] × 100 (1)

where A_1_ was the Abs of the sample, A_2_ was the Abs of the sample only (ethanol instead of DPPH), and A_0_ was the Abs of the control (deionized water instead of sample solution). The bark extract concentration that inhibited 50% of the DPPH radicals (IC_50_) was calculated and expressed as μg/mL. The ascorbic acid was used as a positive control and conducted in parallel. The experiment was carried out in triplicate.

#### 3.5.2. ABTS Radical Scavenging Activity

The ABTS free radical-scavenging activity of the *T. ramosissima* bark extract was determined according to Liu et al. [26]. A mixture (1:1, *v*/*v*) of ABTS (7.0 mM) and potassium persulfate (4.95 mM) was incubated at 25 ℃ overnight in the dark to prepare the fresh stock solution. A working solution was prepared by diluting the stock solution with phosphate buffer solution (pH 7.4, 0.2 M) to obtain an absorbance of 0.70 ± 0.02 at 734 nm. Then, 20 μL of the bark extract solutions at various concentrations (25, 50, 100, 200, 300, 400, and 500 μg/mL) were mixed with 200 μL working solution and kept in the dark for 30 min. The absorbance was then measured at 734 nm. The ascorbic acid was used as a positive control, and the ABTS radical scavenging activity was calculated according to the following equation:

ABTS radical scavenging activity (%) = [1 − (A_1_ − A_2_)/A_0_] × 100 (2)

where A_0_, A_1_ and A_2_ have the same meaning as in Equation (1). The IC_50_ value was also calculated and expressed as μg/mL. All the analyses were performed in triplicate.

#### 3.5.3. Superoxide Anion Radical Scavenging Activity

The superoxide anion radical scavenging of the *T. ramosissima* bark extract was evaluated based on the method of Liu et al. [39], with slight modifications. The reaction mixture contained 1 mL of 50 μM nitro-blue tetrazolium (NBT), 1 mL of 78 μM nicotinamide adenine dinucleotide (NADH), 1 mL of 10 μM phenazine methosulfate (PMS), and 1 mL of the bark extract at different concentrations (25, 50, 100, 200, 300, 400 and 500 μg/mL). After incubation in the dark for 10 min at 37 °C, the absorbance was measured at 560 nm. The ascorbic acid was used as a positive control and triplicate tests were conducted for each sample. The superoxide anion radical scavenging activity was calculated according to Equation (3):

Superoxide anion radical scavenging activity (%) = [1 − (A_1_ − A_2_)/A_0_] × 100 (3)

where A_0_, A_1_ and A_2_ have the same meaning as in Equation (1). The IC_50_ value was calculated and expressed as μg/mL.

#### 3.5.4. Hydroxyl Radical Scavenging Activity

The hydroxyl radical scavenging activity of the *T. ramosissima* bark extract was assayed according to Li [40], with some modifications. The hydroxyl radicals were generated in a Fenton reaction by incubating for 60 min at 37 °C in the presence of 1.0 mM FeSO_4_, 2.0 mM H_2_O_2_, 1.0 mM Ethylene diamine tetraacetic acid (EDTA), 1.0 mM sodium salicylate, 20 mM NaH_2_PO_4_-Na_2_HPO_4_ buffer (pH 7.4), and sample solutions of various concentrations (25, 50, 100, 200, 300, 400, and 500 μg/mL) in a final volume of 5 mL. The solutions of FeSO_4_ and H_2_O_2_ were freshly produced in distilled water just before use. After incubation, the absorbance was measured at 532 nm and all samples were determined in three replicates. The results were calculated according to Equation (4):

Hydroxyl radical scavenging activity (%) = [1 − (A_1_ − A_2_)/A_0_] × 100 (4)

where A_0_, A_1_ and A_2_ have the same meaning as in Equation (1). The IC_50_ value was calculated and expressed as μg/mL.

#### 3.5.5. Reducing Power

The reducing power of *T. ramosissima* bark extract was determined according to the method of Ye et al [41] with several modifications. The sample was dissolved in phosphate buffer saline (PBS) (pH 6.6, 0.2 M) to afford various concentrations (25, 50, 100, 200, 300, 400, and 500 μg/mL). 2.5 mL sample solutions and 2.5 mL of 1% potassium ferricyanide were mixed and incubated at 50 ℃ for 20 min. Then, the mixture was cooled to 25 ℃ and 2.5 mL of 10% trichloroacetic acid was added. The mixture was centrifuged at 650× *g* for 10 min. The supernatant (2.5 mL) was mixed with 2.5 mL of distilled water and 0.5 mL of 0.1% ferric chloride. After thoroughly mixing, the absorbance was measured at 700 nm by a microplate reader, and the ascorbic acid was used as a positive control. Triplicate tests were conducted for each sample, and a higher absorbance indicated a higher reducing power. The reducing power was calculated according to the formula below:

Reducing power = A_1_ − A_2_ (5)

where A_1_ is the absorbance of the sample and A_2_ is the absorbance of the sample only (distilled water instead of ferric chloride). The EC_50_ value is the effective dose of the bark extract yielding an absorbance of 0.5 for reducing power and expressed as μg/mL.

#### 3.5.6. Ferric Reducing Antioxidant Power (FRAP) Assay

The FRAP assay was performed according to Liu et al. [26], with some modifications. Briefly, 0.2 mL *T. ramosissima* bark extract solution at different concentrations (25, 50, 100, 200, 300, 400, and 500 μg/mL) was added to 3.8 mL of FRAP reagent (10 volumes of 300 mM sodium acetate buffer at pH 3.6, 1 volumes of 10.0 mM 2,4,6-tripyridyl-s-triazine (TPTZ) solution, and 1 volume of 20.0 mM FeCl_3_∙6H_2_O solution), and then the mixture was warmed to 37 °C for 30 min in the dark. The absorbance was then measured at 593 nm. The antioxidant activity was calculated from the calibration curve (y = 0.1446x + 0.0478) in the range from 0.15 to 1.50 mM FeSO_4_ with good linearity (R^2^ = 0.9943). The results were expressed as mM FeSO_4_ equivalent/g extract. The ascorbic acid was used as a positive control, and all analyses were performed in triplicate.

### 3.6. Determination of Antimicrobial Activity

#### 3.6.1. Microorganisms

The target bacterial strains used in the study were seven American Type of Culture Collection (ATCC) strains: Gram-positive bacteria including *S. aureus* (ATCC 25923), *L. monocytogenes* (ATCC 13932), *B. cereus* (ATCC 11778), and Gram-negative bacteria including *E. coli* (ATCC 35218), *P. aeruginosa* (ATCC 27853), *S. typhimurium* (ATCC 14028), and *S. castellani* (ATCC 12022). The strains were first grown in Mueller Hinton (MH, Hopebio, Qingdao, China) broth at 37 °C for 24 h prior to seeding onto the MH agar.

For the antifungal activity, four clinical isolates of *Penicillium expansum*, *Aspergillus niger*, *Acremonium strictum*, and *Penicillium citrinum* were first grown on Sabouraud dextrose agar (SDA, HANGWEI, China) plates at 28 °C for 48 h.

#### 3.6.2. Agar Disc Diffusion Method

The Kirby–Bauer agar disc diffusion method was performed to determine the antibacterial and antifungal activity of the *T. ramosissima* bark extract following procedures previously described by Ebani et al [42]. Briefly, isolated colonies were selected to prepare the bacterial inocula in sterile saline solutions to obtain a turbidity equivalent to a 0.5 McFarland standard, approximately 1 to 2 ×10^7^ CFU/mL. Meanwhile, the fungal spores were picked into the sterile saline solution and adjusted to 10^4^ to 10^5^ CFU/mL. An aliquot of 0.1 mL of bacterial (or spore) suspension was spread onto an MH agar plate (SDA for fungi) and then a sterile filter disc with 6 mm diameter (Whatman paper no. 3), which was impregnated with the bark extract of different concentrations (1, 5, 10 mg/mL) in dimethyl sulfoxide (DMSO, Oxoid), was placed on the surface of the media. The plates were incubated at 37 °C for 24 h, and 28 °C for 48 h for fungi, followed by the measurement of the diameter of the growth inhibition zone expressed in millimeters (mm). DMSO was used as negative control, with gentamycin and ketoconazole (Amresco, USA) as positive controls for bacteria and fungi, respectively. All tests were performed in triplicate.

#### 3.6.3. Determination of Minimum Inhibitory Concentration (MIC)

The MIC of the *T. ramosissima* bark extract was determined for each bacterial strain, which was sensitive to the bark extract of the Kirby-Bauer assay, using the microdilution broth method in 96-well microplates. The tests were carried out in MH broth, and a stock solution (20 mg/mL) of the bark extract was prepared in 10% DMSO. An aliquot of this solution was serially diluted (two-fold) with MH broth to obtain a concentration ranging from 20 to 0.15625 mg/mL. After careful mixing, the microplates were incubated at 37 °C for 24 h. The absorbances of the plates at 620 nm were measured with a multiplate reader (SpectraMax M3, Molecular Devices, USA). The MIC value was defined as the lowest concentration of the bark extract at which there was no visible growth of the microorganisms [43]. The results were expressed as mg/mL, and all tests were performed in triplicate.

#### 3.6.4. Determination of Minimum Bactericidal Concentration (MBC)

The MBC was determined by adding 100 μL aliquots of the microplate well contents which did not show any growth in the MIC test, to the MH agar and then incubating at 37 °C for 24 h. The MBC was defined as the lowest concentration of the bark extract which showed no bacterial growth [43].

### 3.7. Statistical Analysis

All analyses were performed in triplicate. The results were expressed as means ± standard deviations. The IC_50_ values were calculated by linear regression analysis. The statistics analysis was performed using SPSS software (version 22.0) by one-way analysis of variance (ANOVA). *p*-value < 0.05 was regarded as significant.

## 4. Conclusions

In the current work, hispidulin and cirsimaritin were first identified from the bark extract of *T. ramosissima* in southern Xinjiang. Hispidulin, cirsimaritin, and isorhamnetin, abundant polyphenolics in the extract, were found at levels of 28.79, 13.35 and 36.91 μg/mg extract, respectively. The bark extract showed satisfying antioxidant activity with a similar DPPH scavenging activity and reducing power to ascorbic acid at 300, 400, and 500 μg/mL. The bark extract exhibited excellent antibacterial activities against foodborne pathogens. *L. monocytogenes*, *S. aureus* and *B. cereus* were sensitive to the bark extract compared with *E. coli*, *P.aeruginosa*, *S. typhimurium* and *S. castellani*. *L. monocytogenes* was the most sensitive bacteria among these foodborne pathogens. Future investigation should focus on the effects of *T. ramosissima* on hazardous substance formation and sensory properties during food processing, as well as the potential health-promoting properties.

## Figures and Tables

**Table 1:** Comparison of TPC and TFC with published data of *Tamarix* family.

*Tamarix* Species	Tested Part	Location	TPC (mg GAE/g)	TFC (mg QE/g)	Reference
*T. ramosissima*	barks	South Xinjiang, China	323.45 ± 21.41	87.32 ± 1.65	This work
*T. gallica*	leaves	South Tunis	34.44 ± 3.40	3.91 ± 0.45	Ksouri et al. [5]
*T. gallica*	flowers	South Tunis	135.35 ± 7.70	12.33 ± 2.10	Ksouri et al. [5]
*T. aphylla*	leaves	South Algeria	199.54 ± 1.60	ND	Mohammedi [9]

Note: The TPC and TFC were presented as mean ± SD. ND means not detected.

**Table 2:** Peak identification of *T. ramosissima* bark extract using UPLC-MS.

Peak No.	Polyphenolic Compounds	Retention Time (min)	Empirical Formula	Calcd *m*/*z*	Obsd *m*/*z* [M + H]^+^	Obsd *m*/*z* [M − H]^+^
1	Quercetin 3-O-glucuronide	4.63	C_21_H_18_O_13_	478.0747	479.0820	477.0676
2	Kaempferol 3-O-glucuronide	5.19	C_21_H_18_O_12_	462.0798	463.0882	461.0802
3	Eriodictyol	6.63	C_15_H_12_O_6_	288.0634	289.0703	287.0581
4	Quercetin	6.77	C_15_H_10_O_7_	302.0427	303.0498	301.0404
5	Naringenin	7.65	C_15_H_12_O_5_	272.0685	273.0753	271.0583
6	Tangeretin	7.72	C_20_H_20_O_7_	372.1209	373.1274	371.1253
7	Kaempferol	7.85	C_15_H_10_O_6_	286.0477	287.0547	285.0405
8	Hesperetin	8.07	C_16_H_14_O_6_	302.0790	303.0856	301.0718
9	Isorhamnetin	9.25	C_16_H_12_O_7_	316.0583	317.0653	315.0510
10	Hispidulin	10.41	C_16_H_12_O_6_	300.0634	301.0705	299.0562
11	Apigenin	10.42	C_15_H_10_O_5_	270.0528	271.0597	269.0427
12	Glycitein	11.90	C_16_H_12_O_5_	284.0685	285.0754	283.0664
13	Cirsimaritin	11.91	C_17_H_14_O_6_	314.0790	315.0863	313.0717

**Table 3:** Concentrations of four representative polyphenolic standards.

Compounds	Concentration (μg/mg)
Isorhamnetin	36.9055
Hispidulin	28.7915
Cirsimaritin	13.3513
Quercetin	4.2065

**Table 4:** Antibacterial activity of *T. ramosissima* bark extracts at different concentrations. Minimum bactericidal concentration (MBC), minimum inhibitory concentration (MIC).

Bacterial Strains		The Extract Concentrations (mg/mL)	Diameter of Inhibition Zones		MIC (mg/mL)	MBC (mg/mL)
			Bark Extract	Gentamycin (10 UI)		
Gram- positive strains	*Staphylococcus aureus*	1	10.60 ± 0.54 ^A^	20.90 ± 0.14	5	15
		5	11.08 ± 0.71 ^A^			
		10	11.16 ± 0.91 ^B^			
	*Listeria monocytogenes*	1	10.14 ± 0.48 ^cAB^	20.30 ± 0.42	5	10
		5	11.18 ± 0.21 ^bA^			
		10	12.26 ± 0.61 ^aA^			
	*Bacillus cereus*	1	9.53 ± 0.62 ^bBC^	18.00 ± 0.00	5	20
		5	9.72 ± 0.16 ^bB^			
		10	10.88 ± 0.30 ^aB^			
Gram- negative strains	*Escherichia coli*	1	9.26 ± 0.65 ^bC^	17.80 ± 0.28	10	25
		5	9.58 ± 0.34 ^bB^			
		10	10.68 ± 0.81 ^aB^			
	*Pseudomonas aeruginosa*	1	8.20 ± 0.51 ^bD^	17.10 ± 0.14	>10	NA
		5	8.85 ± 0.70 ^abC^			
		10	9.38 ± 0.28 ^aC^			
	*Salmonella typhimurium*	1	8.00 ± 0.24 ^bD^	21.75 ± 0.35	>10	NA
		5	8.34 ± 0.66 ^bC^			
		10	9.48 ± 0.52 ^aC^			
	*Shigella castellani*	1	10.65 ± 0.45 ^A^	20.90 ± 0.14	5	15
		5	10.74 ± 0.50 ^A^			
		10	11.16 ± 0.69 ^B^			

Note: Inhibition zone calculated in diameter around the disc (mean ± SD). Different lowercase letters (a~c) within the same bacteria mean significant differences between different concentrations (*p* < 0.05); Different capital letters (A–D) within the same concentration mean significant differences between different bacteria (*p* < 0.05) and no letters indicates no significant difference (*p* > 0.05). NA represented not active. The diameter of disc was 6 mm. Each experiment was done in triplicate.
